# Brachytherapy stents versus conventional plastic stents for the treatment of malignant ureteral obstruction: initial experience

**DOI:** 10.3389/fonc.2025.1625929

**Published:** 2026-01-09

**Authors:** Yipu Li, Chengzhi Zhang, Mengyao Song, Zhanguo Sun, Xinwei Han, Xueliang Zhou, Dechao Jiao

**Affiliations:** Department of Interventional Radiology, The First Affiliated Hospital of Zhengzhou University, Zhengzhou, Henan, China

**Keywords:** ^125^I seeds, brachytherapy, interventional radiology, malignant ureteral obstruction, stent

## Abstract

**Objective:**

To evaluate the feasibility of a newly designed integrated plastic stent loaded with radioactive ^125^I seeds (brachytherapy stent) for the treatment of malignant ureteral obstruction (MUO).

**Methods:**

From May 2018 to May 2023, 30 patients with MUO underwent brachytherapy plastic stent placement (experimental group), while 41 patients underwent conventional plastic stent placement (control group). The primary endpoints were technical success, complications, the local disease response rate (DRR) and the gross haematuria control (GHC) rate; the secondary endpoints were the hydronephrosis Girignon score (HGC) and overall survival (OS).

**Results:**

The rates of technical success (100% vs. 100%), complications (26.7% vs. 14.6%) and HGC (1.5 ± 0.5) vs. (1.5 ± 0.5) in the experimental and control groups were not significantly different (p>0.05). The DRR (93.3% vs. 9.8%) and GHC rates (84.2% vs. 20.8%) of the experimental and control groups at 8 weeks were statistically significant (P<0.05). The OS [28.4 month (95% CI: 24.6–32.3) vs. 19.5 month (95% CI: 17.0–22.0)] of the experimental and control groups was significantly different (P<0.05).

**Conclusion:**

This novel brachytherapy stent is safe and effective for the treatment of MUO, but long term follow up is needed.

## 1 Introduction

Common causes of malignant ureteral obstruction are ureteral carcinoma (UC), bladder carcinoma, cervical carcinoma and retroperitoneal metastases (1, 2). UC accounts for 5%-10% of urinary system tumors, ranking third after kidney and bladder cancer, and has slightly increased in incidence in recent years (3). The standard treatment plan for UC is ipsilateral nephroureterectomy-partial cystectomy on the affected side. The main reason for this treatment is that both the ureter and the bladder develop mesodermal structures, and detached malignant epithelial cells are prone to implantation metastasis in the bladder with the flow of urine (4). Unfortunately, patients who have contraindications to anesthesia, renal insufficiency, a solitary kidney, and subjective refusal of surgical procedures may be recommended for radiotherapy or may only undergo partial placement of D–J tubes to alleviate the obstruction (5, 6). However, prolonged radiotherapy may lead to intestinal or spinal cord injury or retroperitoneal fibrosis, and expensive radiotherapy equipment and high demand for clinical doctors have made such resources difficult to obtain in ordinary grassroots hospitals in China.

Brachytherapy with radioactive ^125^I seeds, as a novel treatment, features a high local cumulative dose to the tumor and causes less damage to the surrounding tissues; this method has been widely used to treat solid malignant tumors such as prostate cancer, esophageal cancer, lung cancer, liver cancer, and glioma and has shown encouraging clinical efficacy (7–11). Can radioactive ^125^I seeds be used to treat MUO? If so, how can the linear ureteral route be matched with ^125^I seeds? To better treat MUO, our center designed a novel brachytherapy plastic stent (BPS) with two cavities: one plays a role in urine drainage due to multiple side holes, while the other can load radioactive ^125^I seeds, which can continuously release γ-rays to some extent to inhibit tumor progression. Thus, such a device can conform to the course of the long strip distribution of the ureter with the aim of “killing two birds with one stone”.

## 2 Materials and methods

### 2.1 Patients

From May 2018 to May 2023, 30 patients with MUO underwent a BPS (experimental group), whereas 41 patients underwent a conventional plastic stent (control group). The inclusion criteria were as follows: (1) 18–80 years of age, (2) evidence of MUO, (3) maximum transverse diameter of the UC <40 mm, (4) estimated life expectancy of more than 3 months, (5) Eastern Cooperative Oncology Group (ECOG) score ≤ 3, and (6) inability or unwillingness to undergo curative surgical treatment. The exclusion criteria were (1) previously received local radiation therapy, (2) severe cardiopulmonary dysfunction (New York Heart Association (NYHA) classification≥3), (3) severe coagulation dysfunction (platelet count ≤30×10^9^/L and prothrombin time > 25 s), and (4) incomplete electronic information. The detailed information of both groups is listed in **Table 1** (Patients characteristics). This study was approved by the ethical committee of our hospital (Clinical Ethics Number: 2018-ky-0302).

**Table 1:** Patients characteristics.

Variables	Brachytherapy group	Control group	P value
	(n=30)	(n=41)	
Age (year old)	72.2 ± 9.0 (56.0-88.0)	71.6 ± 7.5 (58.0-88.0)	0.75
Sex (male/female, n%)	14 (46.7%)/16 (53.3%)	20 (48.8%)/21 (51.2%)	0.86
Gross hematuria (yes/no, n%)	19 (63.3%)/11 (36.7%)	24 (58.5%)/17 (41.5%)	0.68
Diagnosis methods (Fluoroscopy/endoscopy/others, n%)	14 (46.7%)/13 (43.3%)/3 (10%)	24 (58.5%)/13 (31.7%)/4 (9.8%)	0.57
Primary diagnosis (UC/non-UC)	21 (70%)/9 (30%)	27 (65.9%)/14 (34.1%)	0.71
Pathological classification (Low-medium/high differentiation, %)	11 (36.7%)/19 (63.3%)	14 (34.1%)/27 (65.9%)	0.83
Location (high-middle/low ureter)	8 (26.7%)/22 (73.3%)	10 (24.4%)/31 (75.6%)	0.83
Body Mass Index (kg/m^2^)	24.1 ± 2.7 (19.8-30.3)	24.9 ± 2.7 (19.9-30.3)	0.24
Local tumor length (cm)	3.7 ± 0.8 (2.3-5.5)	3.7 ± 0.8 (2.3-5.5)	1.00
Max. diameter (cm)	2.3 ± 0.9 (0.9-3.8)	2.3 ± 0.7 (1.0-3.5)	0.91
ECOG score (0-1/2-3)	16 (53.3%)/14 (46.7%)	19 (46.3%)/22 (53.7%)	0.56
Clinical stage (stage III/IV)	15 (50.0%)/15 (50.0%)	21 (51.2%)/20 (48.8%)	0.92
Girignon score	4.1 ± 0.7 (3-5)	4.2 ± 0.6 (3-5)	0.65
Seed number	16.8 ± 2.3 (12.0-22.0)	–	–
D90 dose (Gy)	46.7 ± 6.5 (37.6-61.5)	–	–
Organ at risk dose (Gy)	3.8 ± 0.5 (2.2-4.5)	–	–
Biochemical indicators	–	–	–
WBC (×10^9^/L)	6.0 ± 1.4 (3.2-8.3)	6.2 ± 1.5 (3.2-9.7)	0.42
Hemoglobin (g/L)	117.7 ± 10.9 (100.0-139.0)	118.4 ± 11.3 (100.0-144.0)	0.78
Alanine aminotransferase (U/L)	38.1 ± 12.1 (24.4-78.7)	37.9 ± 10.7 (22.4-71.9)	0.97
C-reactive protein (mg/L)	8.1 ± 2.8 (2.0-16.0)	8.0 ± 2.1 (4.0-13.0)	0.82
Creatinine (µmol/L)	75.5 ± 18.1 (38.0-112.0)	74.8 ± 17.1 (45.0-106.0)	0.87
Ureanitrogen (mol/L)	6.0 ± 1.6 (2.4-8.4)	6.1 ± 1.5 (3.2-8.5)	0.75

SD, standard deviation; ECOG, Eastern Cooperative Oncology Group; WBC, white blood cell; UC, ureteral cancer.

### 2.2 BPS and ^125^I seeds

**Figure 1:**
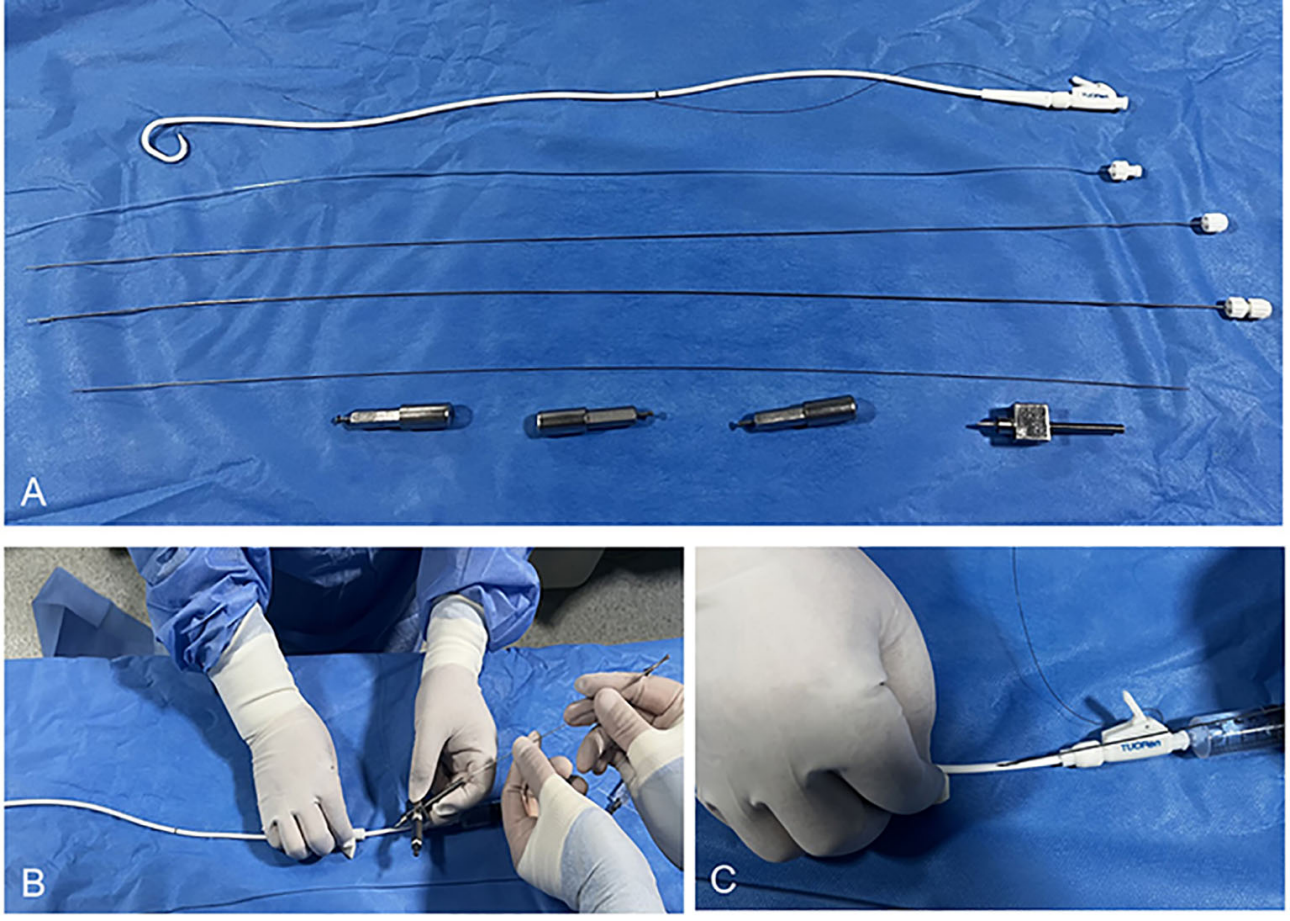
**(A)** The brachytherapy plastic stent is 10F (total length of 55 cm) and has two lumens: one lumen (2–2.2 mm in diameter) serves as the channel for urine drainage with multiple holes, and the other lumen (0.82 mm in diameter) is distributed in parallel and serves as the channel for ^125^I seeds; the opening hole is located at the proximal end of the plastic stent. **(B)** A single seed is inserted through the seed channel one by one. **(C)** A 0.18-inch loach guidewire is inserted into the non-^125^I seed area to prevent seed displacement, and a fixed cap is used to fix the proximal end of the seed channel inlet.

The novelly designed BPSs were produced by Tuoren Medical Device Co., Ltd. (Henan, China) (**Figure 1**). The external diameter of the BPS was 10F (total length of 55 cm), and it had two lumens: one lumen (2–2.2 mm in diameter)served as the channel for urine drainage with multiple holes (distribution length of 40 cm), and the other lumen (0.82 mm in diameter), which was distributed in parallel, served as the channel for ^125^I seeds; the opening hole was located at the proximal end of the BPS. The number of ^125^I seeds needed was calculated according to the formula [length of ureteral stenosis (mm) + 40 (mm)/4.5]. A 0.18-inch loach guidewire was inserted into the non-^125^I seed area to prevent seed displacement, and a fixed cap was used to fix the proximal end of the seed channel inlet (**Figure 1**).

The size of a single ^125^I seed was 0.8×4.5 mm, the seed activity was 0.8 mCi, the average energy was 27–35 keV, the half-life was 59.6 days, the half-value layer was 0.025 mm lead, and the tissue penetration capacity was 1.7 cm. To evaluate the linear ^125^I seed strand cumulative dose, the dose reference point was set to 5 mm from the BPS. The organ at risk (OAR) was set at the abdominal aorta next to the ureter, and the D90 (the dose covering 90% of the gross tumor volume) and OAR doses were calculated by the treatment planning system (TPS, Beijing Astro Technology Co. Ltd.).

### 2.3 Interventional procedures

The patient was prone on the digital subtraction angiography (DSA, Artis-zeego, Siemens Germany) examination table with oxygen and cardiac monitoring. The affected kidney area was disinfected and taped as usual. After local anesthesia with 2% lidocaine, a 21G Chiba needle (Cook, USA) was used to puncture the dilated collection system under ultrasound guidance. A 0.018-inch platinum guidewire was inserted, and a 6F dilator was exchanged and introduced. Through the dilator, a 0.035-inch soft guidewire and a 5F vertebral artery catheter (Cordis, USA) were introduced, both of which cooperated to reopen the obstructed segment of the ureter. The length of the obstructed segment and the number of ^125^I seeds required were recorded and calculated. If there was no evidence of malignant pathology, a DSA-guided forceps biopsy was performed on the ureteral obstruction as follows: an 8F sheath was inserted along with the guidewire, with the head end located above the occluded stenotic segment. Then, biopsy forceps (4.5 mm in diameter and 120 cm in length; Nanjing Micro-Tech Co., Ltd., China) were inserted through the sheath to the beginning of the ureteral stenosis to obtain the sample according to our previous study (12).

**Figure 2:**
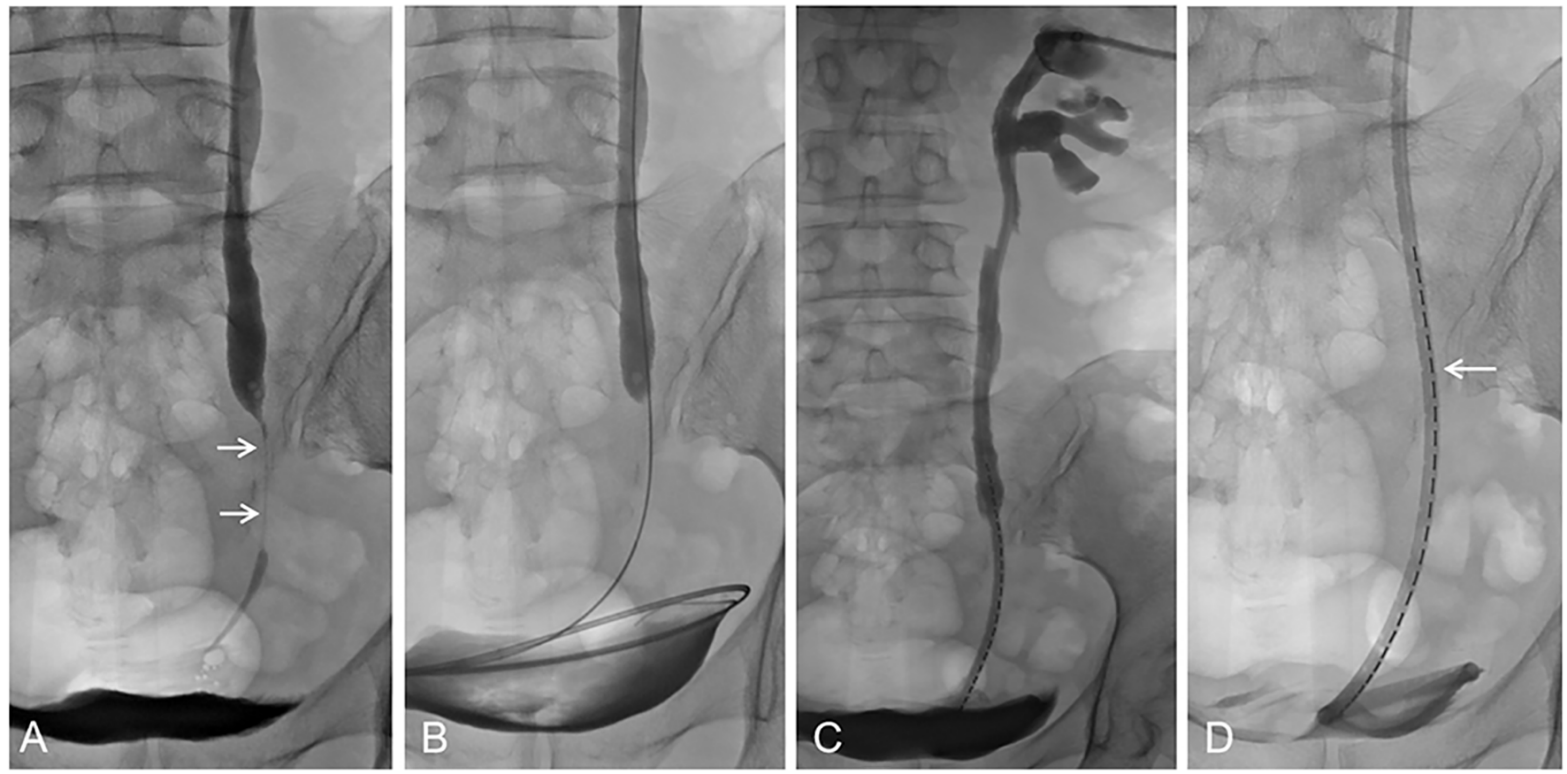
**(A)** Percutaneous transrenal pyeloureterography showing severe stenosis of the lower segment of the ureter (arrow). **(B)** With the assistance of a catheter, the guide wire passes through the ureteral stenosis area and establishes a guide wire pathway. **(C)** The novel BPS loaded with ^125^I seeds advances along the guide wire. **(D)** The local enlarged image shows ^125^I seeds distributed parallel to the urine drainage cavity.

If there was already evidence of malignant pathology (26 people in this study completed endoscopic biopsy before BPS placement), the novel BPS could be placed along the 0.035 inch strengthened guidewire. If it was difficult to pass through the obstructed area, a 5 mm balloon (Bard Company, USA) could be used to expand the stenosis area, the BPS could be placed smoothly, and the side hole of the drainage lumen was crossed over the obstructed area of the ureter. If the patient did not have any infection or fever, the external-internal BPS was closed, and urine was able to pass through the drainage lumen to the bladder (**Figure 2**). All intervention procedures in the control group were the same as those in the experimental group, except that a 10F conventional plastic stent (Cook, USA) was used to cross the ureteral obstruction.

### 2.4 Evaluation and follow-up

**Figure 3:**
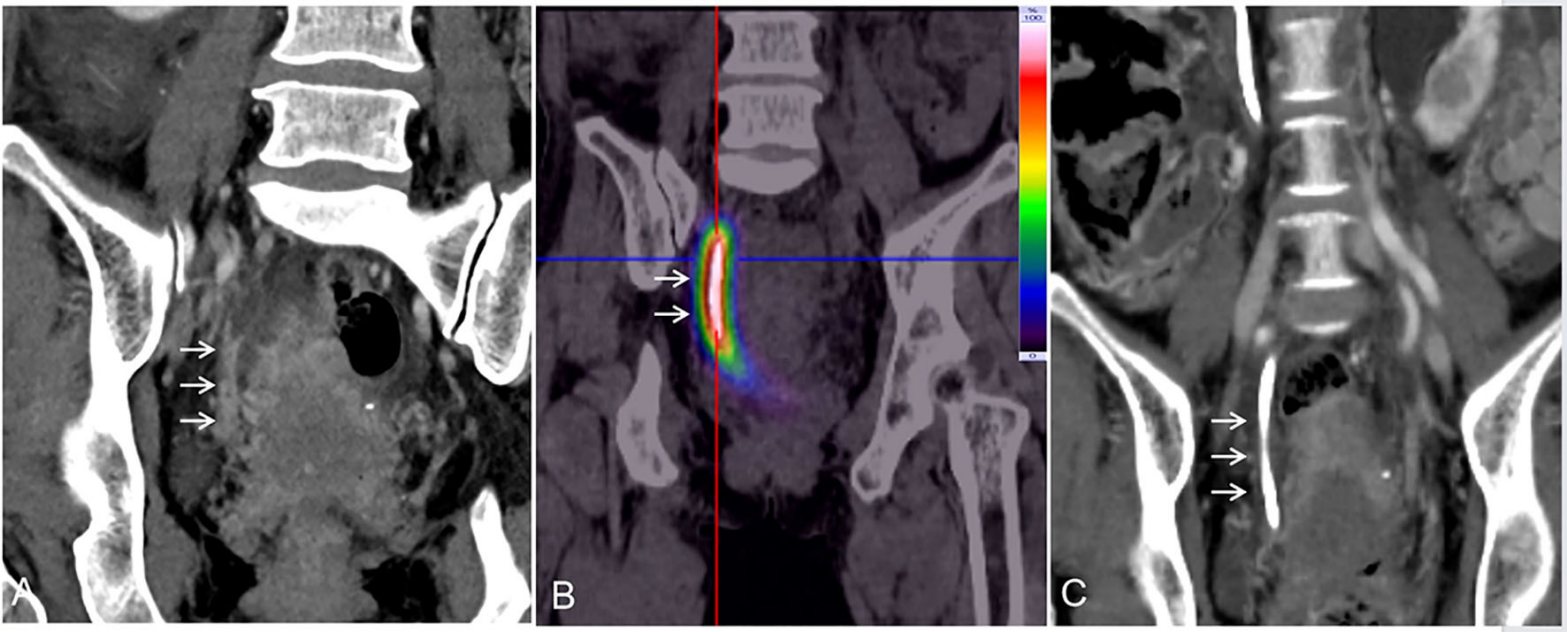
**(A)** Preoperative enhanced CT image showing thickening of the right lower ureteral wall (arrow) and ureteral carcinoma confirmed by forceps biopsy. **(B)** After implantation of this novel BPS, SPECT revealed a local γ-ray distribution (arrow), and the surrounding normal organ radiation dose was extremely low. **(C)** After two months of brachytherapy, the local tumor disappeared (arrow), and the expansion of the collection system disappeared.

Patients underwent SPECT scanning to evaluate the local γ-ray distribution 3 days after BPS placement, and TPS dose verification was performed. The patient’s visible haematuria changes were tracked, a whole abdomen-enhanced CT was performed 8 weeks postoperatively to evaluate the HGC, and the mRECIST criteria were used to evaluate the local tumor response (**Figure 3**). Local disease response rate (DRR) = (complete remission + partial remission + stable disease). The ECOG score was used to evaluate physical status, and the HGC was used for hydronephrosis evaluation. All patients returned to the hospital for review and replacement of D–J tubes 8 weeks after BPS placement. Follow-up appointments were scheduled every 8 weeks thereafter to monitor local and systemic tumor conditions, with detailed records kept. Minor complications were defined as no therapy and no consequence (grade 1) or overnight observation only, no therapy, and no consequence (grade 2). Major complications were defined as those requiring therapy and hospitalization (48 hours, grade 3), permanent adverse sequelae (grade 4), or death (grade 5) (13).

### 2.5 Statistical analysis

All the data were analyzed with SPSS 27.0 software. Continuous data are presented as the means ± standard deviations. Student’s *t* test was used for continuous data, whereas the chi-square test or Fisher’s exact test was used for categorical data. Overall survival (OS) was calculated via the Kaplan–Meier method (Prism 10.1.2, USA). Differences were considered statistically significant at *P* < 0.05.

## 3 Results

**Figure 4:**
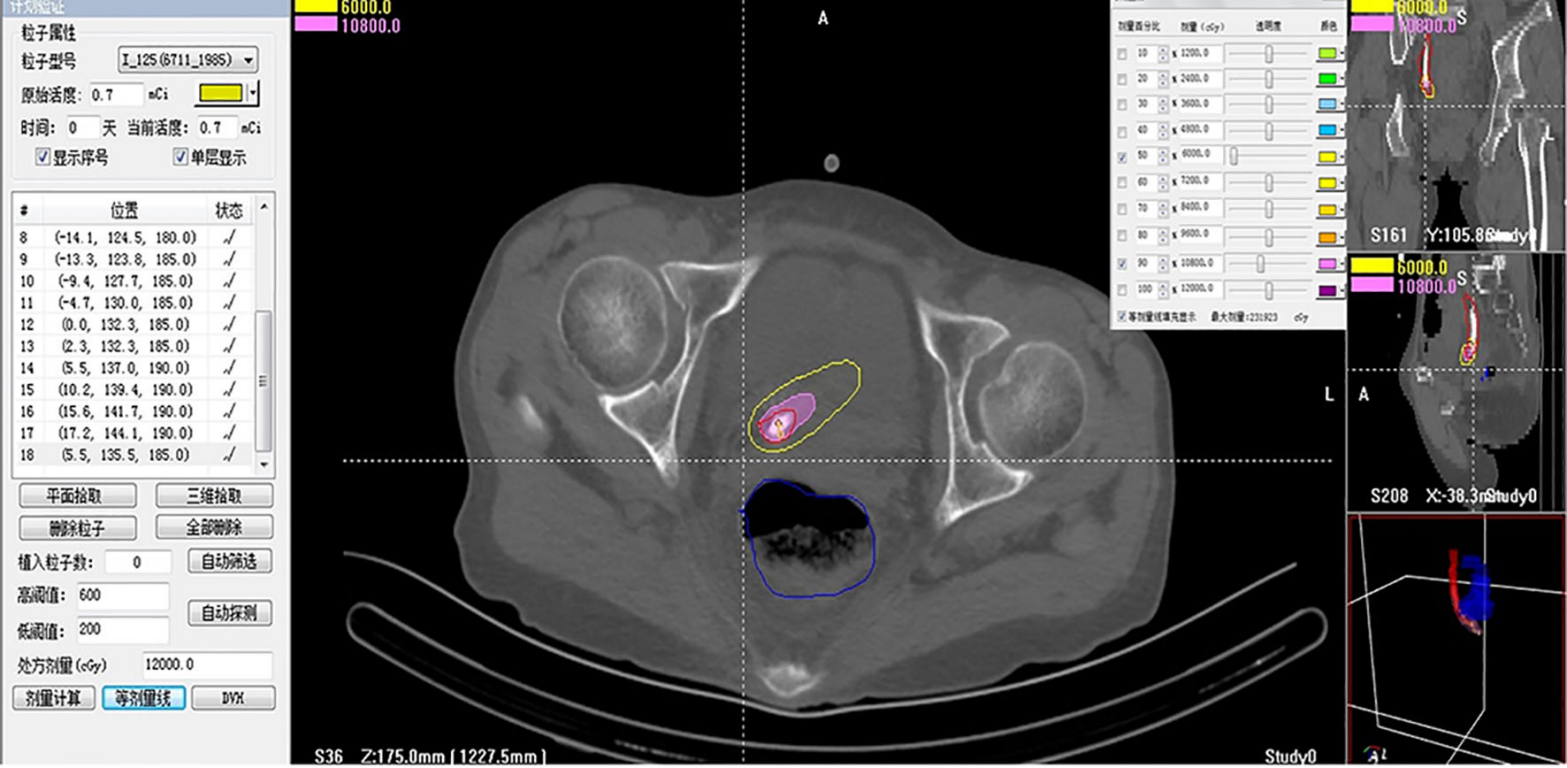
The local cumulative dose 5 mm (dose reference point) from the BPS was calculated via the brachytherapy treatment plan system.

**Figure 5:**
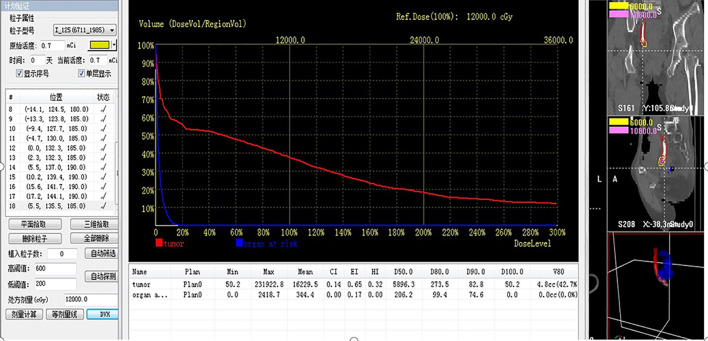
Dose volume histogram of the dose reference point and organ at risk (rectum).

There were no significant differences between the experimental and control groups in terms of characteristics such as age, sex, gross haematuria, diagnosis methods, primary diagnosis, pathological classification, tumor location, body mass index, tumor length, maximum diameter and ECOG score, clinical stage, Girignon grade or main biochemical indicators (all p >0.05). The doses of the reference point and OAR were 46.7 ± 6.5 and 3.8 ± 0.5 Gy, respectively, in the experimental group. Repeated ureterography at 8 weeks showed smooth local ureteral mucosa, but restrictive stenosis was observed in all patients, and 7F D–J tubes were replaced with primary BPSs in the experimental group (**Figures 4**, **5**).

**Figure 6:**
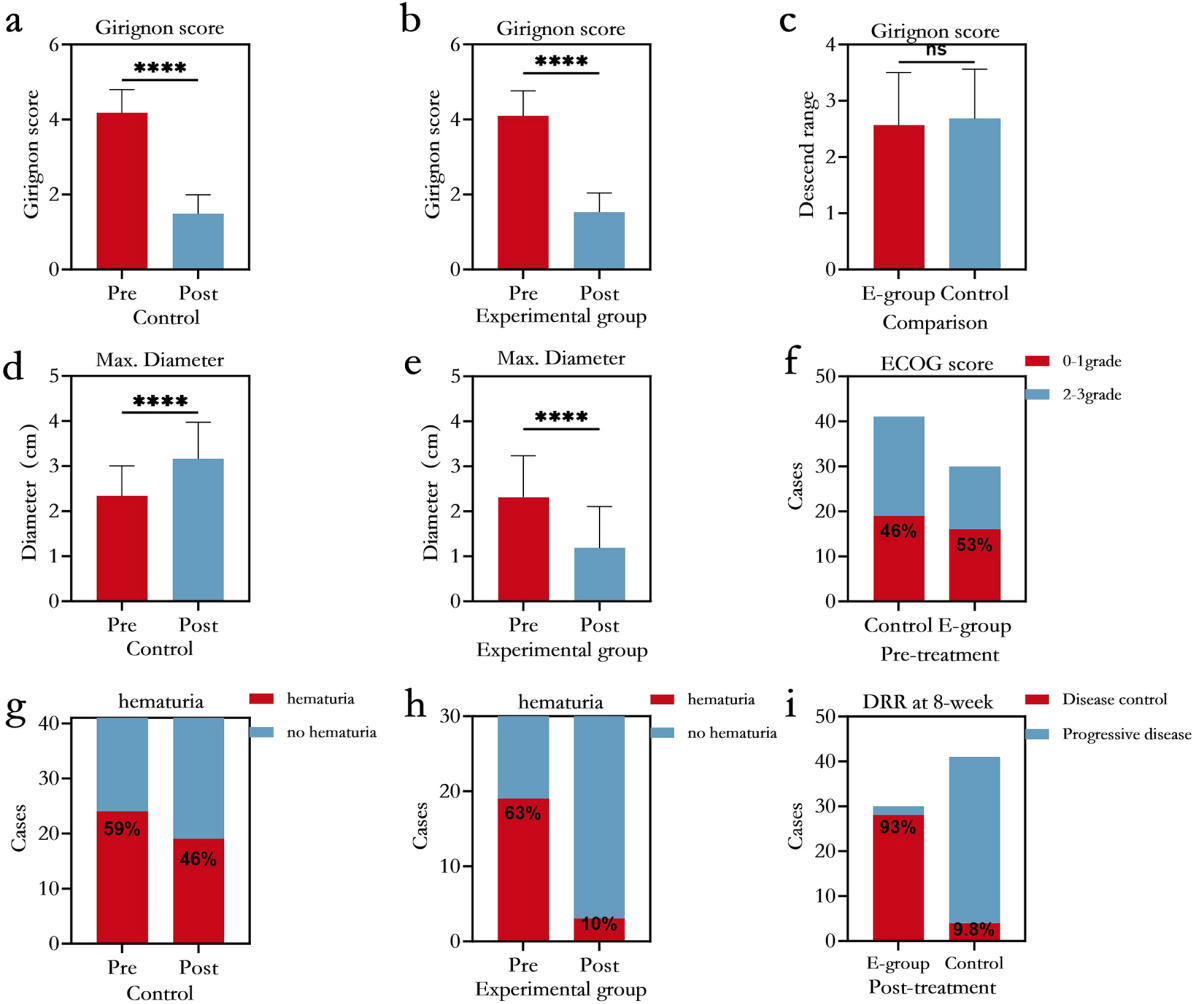
Charts of tumor diameter, Girignon grade, haematuria, Eastern Cooperative Oncology Group (ECOG) score, and local DRR at the 8-week evaluation **(a-c)** Preoperative and postoperative Girignon grades, comparison of the Girignon grade decline in the control and experimental groups. **(d-e)** Preoperative and postoperative maximum tumor diameter in the control and experimental groups. **(f)** ECOG scores of the control group and experimental group before treatment. **(g-h)** Preoperative and postoperative haematuria in the control and experimental groups. **(i)** DRR at 8 weeks in the control group and experimental group after treatment (ns p>0.05, ****p<0.0001).

The technical success rate of both groups was 100%. The average total procedure times were 40.1 ± 9.5 min (range: 26.5–61.2) and 30.4 ± 4.7 min (range: 19.5–41.2) in the experimental and control groups, respectively, which were significantly different (p<0.05). The maximum diameters of the experimental and control groups were 1.2 ± 0.9 and 3.2 ± 0.8 at the 8-week evaluation, respectively, which were significantly different (p<0.05). The local DRR were 93.3% and 9.8% in the experimental and control groups, respectively, which were significantly different (p<0.05). The pre- and posttreatment HGC (4.1 ± 0.7 and 1.5 ± 0.5) in the experimental group and in the control group(4.2 ± 0.6 and 1.5 ± 0.5) were significantly different (p<0.05), whereas the decreases in the Girignon grade were 2.6 ± 0.9 and 2.7 ± 0.9 in the experimental and control groups, respectively, which were not significantly different (p >0.05). There were 19 (63.3%) and 24 patients (58.5%) in the experimental and control groups, respectively, with visible haematuria before treatment, which was not significant (p>0.05), whereas 3 (10.0%) and 19 (46.3%) patients with visible haematuria in the experimental and control groups, respectively, at the 8-week evaluation which was statistically significant (p<0.05) (**Figure 6**).

**Figure 7:**
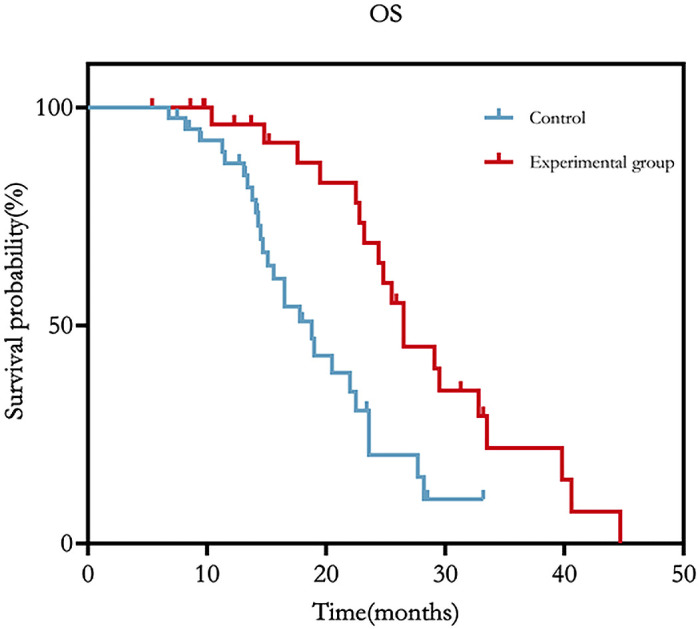
OS of the experimental and control groups.

During the follow-up period, 16 (53.3%) patients in the experimental group and 23 (56.1%) patients in the control group received systemic anticancer treatments, and 17 patients (56.7%) in the experimental group and 40 patients (97.6%) in the control group had local tumor progression during follow-up. During the median follow-up of 17.6 months, 19 and 26 deaths occurred in the experimental and control groups, respectively, and the reasons for death were tumor progression (n=37; 10 and 27 at experimental and control groups) and multiple organ failure (n=8; 3 and 5 at experimental and control groups), respectively. The OS times of the experimental and control groups were 28.4 (95% CI: 24.6-32.3) and 19.5 (95% CI: 17.0-22.0) months, respectively, which were significantly different (p<0.05) (**Table 2**; **Figure 7**).

**Table 2:** Intra- and post-treatment parameters.

Variables	Brachytherapy group	Control group	P value
	(n=30)	(n=41)	
Technical success (n,%)	(30/30)100%	41/41 (100%)	–
Balloon dilation assistance (Yes/no, n%)	6/24 (20.0%/80.0%)	6/35 (14.6%/85.4%)	0.55
Post-treatment gross hematuria (Yes/no, n%)	3/27 (10.0%/90.0%)	19/22 (46.3%/53.4%)	<0.01
Procedure time (min)	40.1 ± 9.5 (26.5-61.2)	30.4 ± 4.7 (19.5-41.2)	<0.01
Hospital stay (day)	5.5 ± 0.8 (4.0-7.0)	5.6 ± 0.8 (4.0-7.0)	0.75
Max. Diameter at 2-month (cm)	1.2 ± 0.9 (0.2-4.2)	3.2 ± 0.8 (1.2-4.6)	<0.01
DRR at 8-week (yes/no)	28/2	4/37	<0.01
Girignon score at 8-week	1.5 ± 0.5 (1.0-2.0)	1.5 ± 0.5 (1.0-2.0)	0.71
Following systemic treatment (Yes/no, %)	16/14 (53.3%/46.7%)	23/18 (56.1%/43.9%)	0.82
Local progression (Yes/no, n%)	17/13 (56.7%/43.3%)	40/1 (97.6%/2.4%)	<0.01
Survival state (live/death)	11/19	15/26	0.99
Overall survival (months95%CI)	28.4 (95%CI24.6-32.3)	19.5 (95%CI17.0-22.0)	<0.01

DRR, Disease response rate; CI, confidence interval.

In terms of complications, catheter obstruction (n=1, Grade 3), fever (n=1, Grade 3), transient aggravated haematuria (n=7, Grade 2), local pain (n=4, Grade 2) and local exudation (n=1, Grade 2) occurred in both groups. However, all major and minor complications were not statistically significant (p>0.05). No patient experienced serious complications, such as ureteral perforation or fatal bleeding. (**Table 3**).

**Table 3:** Complications between the two groups.

Variables	Brachytherapy group	Control group	P value
	(n=30)	(n=41)	
Total (n,%)	8 (26.7%)	6 (14.6%)	0.21
Major complications	2 (6.7%)	0	0.18
Catheter obstruction (n, %)	1 (3.3%)	0	0.42
Fever (n, %)	1 (3.3%)	0	0.42
Minor complications	6 (20%)	6 (14.6%)	0.55
Transient aggravate hematuria (n, %)	4 (13.3%)	3 (7.3%)	0.66
Local pain (n, %)	2 (6.7%)	2 (4.9%)	1.00
Local exudation (n, %)	0	1 (2.4%)	1.00

## 4 Discussion

UC is an unusual type of urological tumor that is often diagnosed due to visible haematuria (14, 15). It accounts for 5% of all primary urological tumors; however, the disease rate of the elderly population has increased according to recent clinical studies (16). In addition to UC, cervical carcinoma, bladder carcinoma, ovarian carcinoma, and retroperitoneal metastases can also invade and compress the ureter, leading to dilation of the collection system above the obstruction and ultimately affecting normal renal function. How to relieve the obstruction of the urinary collection system while simultaneously controlling local tumors has always been a challenging clinical issue. Although surgery is the standard treatment, there are many subjective and objective reasons for surgical contraindications, so minimally invasive therapy is in high clinical demand (17).

Ureteroscopy-guided laser ablation can control intraluminal tumors, but it requires general anesthesia and has a local recurrence rate of more than 35% according to a previous study (18). Traditional radiotherapy is another option, but it is expensive and may cause retroperitoneal fibers; moreover, the dose to surrounding structures were limited by radiotherapy dose and the treatment cycle is relatively long (19). CT-guided radioactive ^125^I seeds implanted into solid tumors via percutaneous technology have been used in China for more than 24 years. New 3D coplanar and noncoplanar templates invented by Chinese scholars have made great progress in increasing clinical efficacy because of their more uniform dose distribution (20). However, the ureter is a long strip with a tubular structure, and it is difficult to implant ^125^I seeds via percutaneous methods. Owing to the inspiration of ^192^Ir intracavitary brachytherapy, our team invented a new BPS with two lumens, one serving as ureteral drainage and another serving as an ^125^I seed channel. This device was implanted into the ureter of normal beagle dogs in our previous experimental study, and although the radiation injury score increased with increasing radiation accumulation, serious complications such as perforation and bleeding did not occur in any of the animals (21). After the safety of ^125^I seed brachytherapy for normal ureters was verified, how to finish clinical ^125^I seed implantation became a practical issue. This pilot study first evaluated the feasibility of ^125^I brachytherapy for MUO.

The ureteral procedures were completed under local anesthesia with a technical success rate of 100% and an average operation time of only 40.1 minutes for the experimental group. Complications did not significantly differ between the two groups, which means that compared with conventional plastic stent, the novel BPS does not increase obvious complications. The reference point dose was 46.7 Gy, which may not meet conventional irradiation dose requirements, suggesting that this method may be palliatively suitable for tumors with a diameter of less than 2 cm. In the future, the stent can be designed as a three-lumen structure with two lumens containing radioactive ^125^I seeds to increase local radiation dose accumulation. This is the future research direction of the team. The OAR (blood vessel) received only 3.8 Gy, which fully utilized the ^125^I brachytherapy, and the surrounding normal tissues were protected to the greatest extent. The local DRR of the experimental group was 93.3%, which was greater than that of the control group and increased by 83.5%, indicating satisfactory local tumor control. The patient’s HGC decreased, and CT revealed that the hydronephrosis above the obstruction had disappeared, indicating that the newly designed BPS did not affect ureter drainage function. The GHC rate after ^125^I seed brachytherapy (84.2% vs. 20.8%) was greater than that of the control group, and the accumulation of the local dose, to a certain extent, had an obvious inhibitory effect on ureteral mucosal tumors, which stopped mucosal bleeding. As expected, a BPS loaded with ^125^I seeds can achieve both urine drainage and brachytherapy.

In our study, we found that after 8 weeks of brachytherapy, all patients’ local contrast images still revealed obstruction in the ureteral region. However, the mucosal surface was smooth, which was consistent with previous animal experiments by our team, and this scarring stenosis is believed to be a result of brachytherapy. To address this issue further, all patients were regularly provided with 7F D–J tube replacement after brachytherapy was discontinued to prevent renal atrophy. During the follow-up period, local progression was observed in 17 patients, which requires continued increased attention. Methods of identifying tumor remnants still need further study, and routine ureteroscopy may be a good solution.

The experience on the newly designed BPS was summarized as follows. (1) The cross-section of the BPS is 10F, and balloon angioplasty can be used to assist in improving the ease of tube insertion. (2) Some patients had to switch from internal-external to external drainage because of local injury after forcible insertion of the 10F drainage tube, which led to slight aggravation of short-term bleeding and causing the lateral holes of the tube to be blocked. (3) The end of the BPS is designed as a three-way tube at the skin. Although it is equipped with a fixation patch, it still affects the ability of patients to sleep in the supine position. An integrated D–J tube design in the future could better meet clinical needs. (4) Placing the BPS retrogradely via cystoscopic guidance may better reflect the core concept of minimally invasive treatment. (5) Fifty-six percent of the patients in the experimental group experienced local progression at follow-up visits. In particular, some patients developed fibrosis-like hyperplasia after local ureteral irradiation, which could not be differentiated from tumor remnants. (6) Considering the low standard of ^125^I seed protection in the clinic, this technique is suitable for rural hospitals and holds a promising future.

Recently, novel integrated BPSs loaded with ^125^I seeds for the treatment of MUO have shown marked effects. However, there are still shortcomings, such as not a prospective randomized controlled trial, but rather a result of alternating allocation by clinical physicians, the program is still in the stage of accumulating early experience, so there is still a need to increase the sample size, prolong the follow-up time, and design more open controlled clinical studies in the future so that more objective clinical results can be obtained.

In conclusion, the novel integrated BPS loaded with ^125^I seeds is safe and effective in patients with MUO, and the OS and local DRR are significantly better than those in the conventional stent group.

## Data Availability

Due to the nature of this research, participants of this study did not agree for their data to be shared publicly, so supporting data is not available. Requests to access the datasets should be directed to Dechao Jiao, Department of Interventional Radiology, the First Affiliated Hospital of Zhengzhou University, No.1 East Jianshe Road, Zhengzhou 450052, Henan, P.R. China. Email: jiaodechao007@126.com.
